# Navigating the thymic landscape through development: from cellular atlas to tissue cartography

**DOI:** 10.1038/s41435-024-00257-8

**Published:** 2024-02-10

**Authors:** Dörte Symmank, Felix Clemens Richter, André F. Rendeiro

**Affiliations:** 1 Department of Dermatology, Medical University of Vienna, Vienna, 1090 Austria; 2 Institute of Hygiene and Applied Immunology, Department of Pathophysiology, Infectiology and Immunology, Medical University of Vienna, Vienna, 1090 Austria; 3 CeMM Research Center for Molecular Medicine of the Austrian Academy of Sciences, Vienna, 1090 Austria

**Keywords:** Thymus, Next-generation sequencing

The thymus plays a central role in the development and education of T cells. Studying the human thymus is notoriously challenging due to limited access to healthy sample tissue and difficulties in tissue processing. Although single cell sequencing identified more than 40 different cell states, encompassing majorly T cells, thymic fibroblasts, and thymic epithelial cells (TECs) [1]. The thymus is separated into distinct individual lobules, each further divided into two distinct zones based on their cellular density. In the cortex, cortical TECs (cTECs) assist early thymocyte development, which produces CD4^+^ CD8^+^ double positive cells [2]. After migrating to the medulla, tissue-restricted antigen presenting medullary TECs (mTECs) mediate the negative selection process, thereby eliminating strongly self-reactive T cells from the thymus. Additionally, secondary structures, like Hassall’s Corpuscles (HCs) in the medulla and the cortico-medullary junction (CMJ) are suggested to contribute to T cell tolerance and egress, respectively. The vast diversity of stromal cells, along with other hematopoietic cells, creates a highly specialized microenvironment. Despite the broad efforts to characterise the cellular composition of the thymus during its development [3–5], the spatial distribution of these cells in the thymus and how they facilitate the maturation of conventional T cell over the time remained largely unknown.

By combining a powerful ensemble of orthogonal state-of-the-art single-cell assays, the preprint by Yayon, Kedlian, Boehme et al. set out to address these issues [6]. Central to their approach is an anatomically principled data harmonization strategy. To facilitate good comparability between different assays and to account for the specific morphology of the lobule and its 3D complexity, they defined cells by their relative positions within the lobule. Using the idea of defining landmark structures as anchor points, the authors were able to create a continuous cortico-medullary axis (CMA). Here the relative positions of cells are calculated by their weighted distances to boundaries defined by key anatomical landmarks, thereby creating a continuous and finely detailed spatial framework within the tissue structures. Through modelling the spatial dimension of the thymus in a continuous manner, the resulting map allows the authors to identify transcriptional profiles and cellular populations distinct to each region and thus deduce their functionality.

Employing this map, the authors demonstrate that maturating T cells showed an overall similar spatial developmental trajectory in both foetal and paediatric thymus. Expectedly, double negative and positive immature T cells were predominantly located in the cortex, while mature T cells were enriched in the medulla. Cytokine expression showed comparable spatial compartmentalisation across different developmental stages. The study verified spatially distinct distribution between known chemokine ligands in the cortex (e.g., CXCL12 [7]) and medulla (e.g., CCL21 [8]), but also revealed cytokines changing their local distribution upon thymic development. These include IL-34, IL1R, CX3CL1, IL33, SPP1 which could suggest distinct involvements of these cytokines for T cell maturation in the thymus during development. The precise function of this spatially restricted expression remains unknown.

Beyond the immune compartments, the authors locate a putative progenitor population of TECs, so called medullary-cortex TEC (mcTEC) progenitors in different locations of the thymus. mcTEC were found closer to capsular zones, while mcTECs and vessels were closer to the CMJ in the paediatric thymus. This spatial association, which mirrors the distribution of capillaries and lymphatic vessels in the foetal thymus, could suggest that the vascular localisation proves a niche to mcTEC progenitors.

Previous reports have suggested that HCs may be involved in the selection of regulatory T cells (Tregs) egressing from the thymus [9]. Nevertheless, the role of this epithelial cell aggregate within the thymic medulla remains ill-defined. Applying their spatially aware analysis methods, the authors observed high concentration of differentiated mTECs (referred to as mTECIII) with high expression of peripheral tissue antigens. This data points towards an important additional layer of T cell education by ensuring development of tolerance of T cells (e.g. Tregs) towards self-antigens specifically expressed in barrier tissues such as skin and mucosa. Previously, thymic stromal lymphopoietin expression of the HCs was suggested to introduce dendritic cells, which consequently provides important survival signals to developing thymic Tregs. The spatial enrichment and possible interaction of CD11c^+^ DCs and FOXP3^+^ Tregs with HC-associated mTECs would reinforce this hypothesis, but further investigation is needed [9].

Lastly, the authors revealed a distinctive migratory pattern of T cells committed towards CD4 or CD8 lineages between the cortex and medulla. CD8 committed T cells linger in the cortex longer and only depart from the cortex when they reach a (semi)mature stage. In contrast, CD4^+^ T cells move to the medulla earlier. The authors correlate these migratory behaviours with a distinctive utilization of chemokine receptors seen on the transcriptional and protein levels. They pinpoint CCR4 as the critical chemokine receptor guiding CD4-committed T cell to exit from the cortex, while CXCR3 was identified as the key for CD8-committed T cell egress. This seems to fit previous publications which show that CCR4 is crucial for the medullary entry of CD4-committed thymocytes [10].

Overall, Yayon, Kedlian, Boehme et al. demonstrate the importance of analytical methods for spatial and single-cell data grounded on anatomical principles to gain deeper understanding into the organisation of the thymus during different developmental stages. Furthermore, this study serves as a blueprint to homogenise spatial and single cell data to overcome limitations in resolution and sample acquisition. On the biological level, the presented data enhances the understanding of the spatial organization of different microenvironments throughout the development of the thymus. It spurs new biological questions with regards to the involvement of, for example spatially restricted cytokine expression in T cell development as well as the potential impact of HC-associated mTECs in thymocyte education (Fig. 1).

**Fig. 1:**
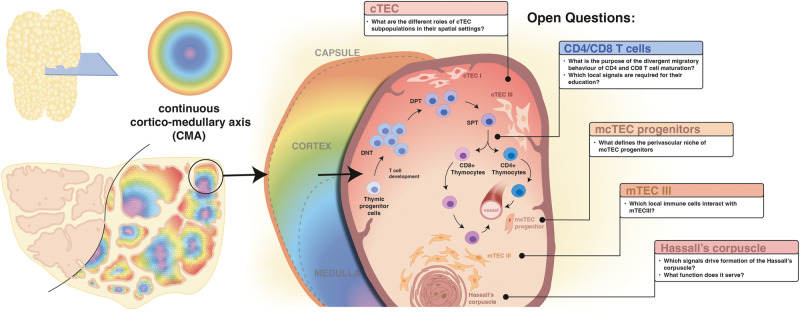
Spatial transcriptomic analysis of thymus driven by anatomically grounded principles open novel questions on the organ’s development. Applying the continuous cortico-medullary axis to the thymus section enables the relative positioning of cells within the lobule, facilitating the observation of their development and movement. Classically, common lymphoid progenitor cells enter the thymus and commit towards their T cell lineage. Thymocytes then progress to rearrange their beta chain in their double negative thymocyte (DNT) state. Upon passing this first checkpoint, the cells upregulate surface expression of CD4 and CD8, thereby entering their double positive thymocytes (DPT) state. Upon subsequent positive and negative selection, single positive thymocytes (SPT) will eventually emerge from the thymus. The preprint adds a new perspective on how developing T cells interact with a multitude of stromal cells, including the cortical thymic epithelial cell (cTEC) in the cortex and medullary thymic epithelial cell subsets (mTEC) in the medulla. Moreover, it highlights the close association of the Hassall’s Corpuscle with mTEC III and the medullary-cortex TEC (mcTEC) progenitors with their perivascular niche. This study sparks a series of new questions, which may provide further mechanistic spatial insights into thymocyte development.

## References

[1] Park JE, Botting RA, Domínguez Conde C, Popescu DM, Lavaert M, Kunz DJ (2020). A cell atlas of human thymic development defines T cell repertoire formation. Science.

[2] Bosticardo M, Notarangelo LD (2023). Human thymus in health and disease: Recent advances in diagnosis and biology. Semin Immunol.

[3] Lavaert M, Liang KL, Vandamme N, Park JE, Roels J, Kowalczyk MS (2020). Integrated scRNA-Seq Identifies Human Postnatal Thymus Seeding Progenitors and Regulatory Dynamics of Differentiating Immature Thymocytes. Immunity.

[4] Bautista JL, Cramer NT, Miller CN, Chavez J, Berrios DI, Byrnes LE (2021). Single-cell transcriptional profiling of human thymic stroma uncovers novel cellular heterogeneity in the thymic medulla. Nat Commun.

[5] Suo C, Dann E, Goh I, Jardine L, Kleshchevnikov V, Park JE (2022). Mapping the developing human immune system across organs. Science.

[6] Yayon N, Kedlian VR, Boehme L, Suo C, Wachter B, Beuschel RT, et al. A spatial human thymus cell atlas mapped to a continuous tissue axis. Immunology; 2023. Available from: http://biorxiv.org/lookup/doi/10.1101/2023.10.25.562925. 10.1038/s41586-024-07944-6 PMC11578893 39567784

[7] Plotkin J, Prockop SE, Lepique A, Petrie HT (2003). Critical Role for CXCR4 Signaling in Progenitor Localization and T Cell Differentiation in the Postnatal Thymus. J Immunol.

[8] Kozai M, Kubo Y, Katakai T, Kondo H, Kiyonari H, Schaeuble K (2017). Essential role of CCL21 in establishment of central self-tolerance in T cells. J Exp Med.

[9] Watanabe N, Wang YH, Lee HK, Ito T, Wang YH, Cao W (2005). Hassall’s corpuscles instruct dendritic cells to induce CD4+CD25+ regulatory T cells in human thymus. Nature.

[10] Hu Z, Lancaster JN, Sasiponganan C, Ehrlich LIR (2015). CCR4 promotes medullary entry and thymocyte–dendritic cell interactions required for central tolerance. J Exp Med.

